# A Cassette Containing Thiostrepton, Gentamicin Resistance Genes, and *dif* sequences Is Effective in Construction of Recombinant Mycobacteria

**DOI:** 10.3389/fmicb.2017.00468

**Published:** 2017-03-24

**Authors:** Julius Mugweru, Gaelle Makafe, Yuanyuan Cao, Yang Zhang, Bangxing Wang, Shaobo Huang, Moses Njire, Chiranjibi Chhotaray, Yaoju Tan, Xinjie Li, Jianxiong Liu, Shouyong Tan, Jiaoyu Deng, Tianyu Zhang

**Affiliations:** ^1^State Key Laboratory of Respiratory Disease, Guangzhou Institutes of Biomedicine and Health, Chinese Academy of SciencesGuangzhou, China; ^2^University of Chinese Academy of SciencesBeijing, China; ^3^Key Laboratory of Biotechnology of Antibiotics, Ministry of Health, Institute of Medicinal Biotechnology, Chinese Academy of Medical Sciences and Peking Union Medical CollegeBeijing, China; ^4^State Key Laboratory of Respiratory Disease, Department of Clinical Laboratory, Guangzhou Chest HospitalGuangzhou, China; ^5^State Key Laboratory of Virology, Wuhan Institute of Virology, Chinese Academy of SciencesWuhan, China

**Keywords:** mycobacteria, selection marker, thiostrepton, gentamicin

## Abstract

The genetic manipulation of *Mycobacterium tuberculosis* genome is limited by the availability of selection markers. Spontaneous resistance mutation rate of *M. tuberculosis* to the widely used kanamycin is relatively high which often leads to some false positive transformants. Due to the few available markers, we have created a cassette containing thiostrepton resistance gene (*tsr*) for selection in *M. tuberculosis* and *M. bovis* BCG, and gentamicin resistance gene (*aacC1*) for *Escherichia coli* and *M. smegmatis* mc^2^155, flanked with *dif* sequences recognized by the Xer system of mycobacteria. This cassette adds to the limited available selection markers for mycobacteria.

## Introduction

Gene manipulation in mycobacteria is performed using a limited number of selection markers. Mycobacteria are naturally resistant to many antibiotics and requires use of stable drugs with low frequency of spontaneous resistance for selection, hence limiting the alternative choices (Parish and Brown, 2008). The combined use of multiple markers enables more versatile genetic modifications, including the stable maintenance of multiple plasmids and inactivation of multiple genes (Wada et al., 2016).

Aminoglycoside phosphotransferase (*aph*) genes, conferring resistance to kanamycin (KAN), were the first to be used as selection markers in mycobacteria (Snapper et al., 1988) owing to their stability over the extended periods of incubation for slow-growing mycobacteria. However, their utility is limited by emergence of spontaneous resistance, albeit at low frequencies (Hatfull, 1996). Unlike fast-growing bacteria, slow-growing mycobacteria have a single rRNA operon (Suzuki et al., 1987) which is more prone to mutations conferring resistance to agents such as KAN (Bottger, 1994). Besides, selection using KAN in *Mycobacterium w* and *Mycobacterium vaccae* has not been achieved. Radford and Hodgson (1991) first reported the use of hygromycin (HYG) resistance gene (*hyg*) as a selection marker in *M*. *smegmatis* and *M. bovis* BCG in 1991. Since then, it has been used in other mycobacteria. The use of *hyg* provides a marker gene which does not provide cross resistance to clinically useful drugs (Garbe et al., 1994). It offers an improved transformation frequency over KAN and is probably more efficiently expressed in mycobacteria than the *Escherichia coli*-derived aminoglycoside phosphotransferase genes conferring KAN resistance (Garbe et al., 1994). The use of apramycin as a selection marker in both slow- and fast-growing mycobacteria was first reported by Paget and Davies in 1996, following its disapproval for clinical use in humans. However, its utility is limited by the acetylation of other closely related aminoglycoside such as KAN (Davies and O‘Connor, 1978; Consaul and Pavelka, 2004), and low transformation efficiencies. β-lactam-based selection markers such as the ampicillin resistance gene *amp*^r^ are not useful in mycobacteria since they contain endogenous β-lactamases that confers natural resistance to penicillins (Hatfull, 1996).

Other past explorations have included resistance to chloramphenicol (Das Gupta et al., 1993), but have been limited due to poor stability and high rates of spontaneous mutations, hence unsuitable for slow-growing mycobacteria. Streptomycin, sulfonamide (Gormley and Davies, 1991) and mercury salts (Baulard et al., 1995) have also been explored as possible selectable markers, but to date, KAN, HYG, and GEN resistance genes remain the often widely exploited selectable markers in mycobacterial genetics.

Owing to the limited number of markers and their disadvantages, we hence sought to explore use of methyl-accepting chemotaxis protein I, a serine sensor receptor *tsr* gene conferring resistance to thiostrepton (TSR), as a selectable marker in *M. tuberculosis* and *M. bovis* BCG. TSR, a thiazole antibiotic, was first isolated and characterized from *Streptomyces azureus* (Cundliffe, 1971) in 1954 at the Squibb Institute (Pagano et al., 1956) and is used in veterinary medicine to treat mastitis, and as a topical agent for dogs. However, it has only found limited applications due to its poor solubility and toxicity (Kuiper and Conn, 2014).

Thiostrepton inhibits protein translation by firmly binding to the complex formed by 23S rRNA and ribosomal protein L11 in bacterial ribosomes (Cannon and Burns, 1971; Cundliffe, 1971). The *tsr* gene encodes an RNA methyltransferase that prevents TSR from binding to ribosomes by 23S rRNA methylation (Thompson et al., 1982). The *tsr* confer total resistance to TSR and thus has been the selection marker of choice in many of the *Streptomyces* spp. cloning vectors (Thompson et al., 1980).

In a recent study on ovarian cancer cell lines, Westhoff et al. (2014) demonstrated that when TSR is used in combination with the standard paclitaxel/cisplatin chemotherapy, it decreases Forkhead box M1 (FOXM1) gene expression besides showing an enhanced synergistic cytotoxicity in ascites cells from platinum-resistant patients. In addition, Wada et al. (2016) also demonstrated *tsr* as a viable selection marker for the thermophilic *Geobacillus kaustophilus* besides demonstrating accurate selection as a single copy in *Streptomyces* strains.

However, only scanty data showed that TSR is active against *M. tuberculosis* (Vermeulen and Wu, 2004; Lougheed et al., 2009) in drug testing.

## Materials and Methods

### Strains, Media, and Culture Conditions

*Escherichia coli* DH5α was grown at 37°C in Luria-Bertani (LB) broth and agar. *M. tuberculosis* H37Rv, autoluminescent *M. tuberculosis* H37Ra (Yang et al., 2015), *M. bovis* BCG Tice and *M. smegmatis* mc^2^155 and their recombinants were grown in Middle Brook 7H9 broth (Becton Dickinson, USA) supplemented with 10% oleic acid albumin dextrose catalase (OADC, Becton Dickinson) and 0.05% tween80, or on solid Middle Brook 7H11 medium (Difco) supplemented with 10% OADC. On agar plates, *M. tuberculosis* H37Rv, *M. bovis* BCG Tice and *M. avium* were incubated for 4–5 weeks, and *M. abscessus* GZ002 and *M. smegmatis* mc^2^155 were incubated for 3–4 days in 37°C.

Thiostrepton and GEN were purchased from Sigma–Aldrich (China) and dissolved in dimethyl sulfoxide (DMSO) and double distilled water, respectively. GEN 20 and 5 μg/mL was used for selection of *E. coli* and *M. smegmatis* mc^2^155, respectively, and TSR 5 and 10 μg/mL of both *M. tuberculosis* H37Rv and *M. bovis* BCG Tice. LB broth was augmented with 170 μg/mL chloramphenicol Sigma–Aldrich (China).

### Drug Susceptibility Testing

We first tested the potential of TSR as a selection antibiotic for *M. tuberculosis* up to a final concentration of 10 μg/mL in liquid culture of autoluminescent *M. tuberculosis* H37Ra (AUlRa) (**Table 1**) as previously described (Zhang et al., 2012). Briefly, 2 mL of AUlRa was inoculated in 50 mL 7H9 plus OADC and tween80 with shaking at 37°C to mid log phase (OD_600_ = 0.6–0.8) in a flask and then diluted to appropriate concentrations. Drugs (5 μL/drug) were added into the 1.5 mL vial, mixed with 195 μL AUlRa and incubated at 37°C. Controls using 195 μL AUlRa and DMSO (5 μL) or 195 μL AUlRa and water (5 μL) tubes were included. Relative light measurements (RLUs) were monitored starting day 0, day 1, day 3, and day 5 using GloMax 20/20 Luminometer (Promega).

**Table 1:** List of plasmids and strains used in the study.

Strains/plasmids	Relevant characteristic(s)^a^	Source or reference
*Escherichia coli* DH5α	General-purpose cloning strain; F^-^ (ϕ80d *lacZ*Δ*M15*) ΔD (*lacZYA-argF*) *U169 deoR recA1 endA1 hsdR17 glnV44 thi-1 gyrA96 relA*	Hanahan, 1983
*Mycobacterium smegmatis* mc^2^155	Highly transformable derivative of ATCC^a^ 607	Snapper et al., 1990
*M. tuberculosis* H37Rv	Widely used virulent laboratory *M. tuberculosis* strain, ATCC^a^ 27294	Zhang et al., 2010
*M. tuberculosis* H37Ra	Selectable marker-free autoluminescent *M. tuberculosis* H37Ra	Yang et al., 2015
*M. bovis* Tice	The live attenuated TB vaccine	Zhang et al., 2010
*M. avium*	Clinical isolate from Guangzhou chest hospital and verified by PCR	Guo et al., 2016
*M. abscessus* GZ002	Clinical isolate from Guangzhou chest hospital with profile of lysine acetylation that shares similarities with *M. tuberculosis*	Guo et al., 2016
p60luxN	p60lux truncated with 18 bp at the 3- of *hsp*60 promoter to remove the six amino acid for fusion expression and introduced at the ATG of *Nde*I as the initiation codon	Liu et al., 2015
p60Gm	0.543 kb *aacC1–gentamicin-(3)-N-acetyltransferase* from *Pseudomonas aeruginosa* plasmid R1033 transposon Tn1696 cloned adjacent to mycobacterial *hsp60* promoter into the *Nde*I–*Pst*I sites of p60luxN, episomal	This study
p60GTE	0.8 kb *tsr* fragment cloned adjacent to *aacC1* on the *Pst*I–*Xba*I sites of p60Gm (*hsp60*–*aacC1*–*tsr* cassette), episomal	This study
pUCDHmke = pTYdHm	Amp^R^, Hyg^R^, *E. coli* high copy number cloning vector bearing the *dif-*Ω*HYG-dif*, episomal	Yang et al., 2014
pUCDGT	*dif-*Ω *hsp60*–*aacC1*–*tsr–dif* cassette cloned into the *Xba*I site of pTYdHm replacing the Hyg^R^ gene, episomal	This study
pMH94	pUC119 carrying KANr from Tn9O3 and *attp-int* cassette from L5 mycobacteriophage at *Sal*I-*Sal*I, integrative	Lee et al., 1991
p60GTI	*dif-*Ω *hsp60*–*aacC1*–*tsr–dif* cassette cloned into the *Hin*dIII site of plasmid pMH94 replacing the Km^R^, *E. coli*–mycobacterial shuttle vector bearing the *attP*:*int* fragment, integrative	This study
pPR27	*E. coli*–mycobacterial shuttle vector, oriM, temp^S^, sacB, xylE, GEN^R^ episomal	Pelicic et al., 1997
pIJ6902	Am^R^, TSR^R^ integrative	Huang et al., 2005

***^*a*^**Abbreviations for resistance phenotypes: Amp^*R*^, ampicillin; Km^*R*^, kanamycin; Hyg^*R*^, hygromycin; Gm^*R*^, gentamicin; Am^*R*^, apramycin; TSR^*R*^, thiostrepton; temp^*S*^, temperature sensitivity; ATCC, The American Type Culture Collection; *dif*, the action site of the XerCD recombinase.*

Susceptibilities of *M. tuberculosis* H37Rv, *M. bovis* BCG Tice, *M. avium* and *M. abscessus* GZ002 to TSR were performed using mid log phase high titer (>10^7^ CFU/plate) cultures on 0, 2, 20, and 100 μg/mL Middle Brook 7H11 TSR agar plates.

Minimum inhibition concentration (MIC) was defined as the lowest concentration of a drug inhibiting 99% of bacterial growth (Zhang et al., 2010). The MIC values for wild-type and recombinant mycobacteria were detected on Middle Brook 7H11 agar plates containing different concentrations of TSR (0–160 μg/mL) and GEN (0–100 μg/mL).

### General DNA Techniques

Polymerase chain reaction (PCR) amplification reactions were performed with pfu DNA polymerase (Takara). The PCR products and plasmids were analyzed by electrophoresis in agarose gels and purified using a DNA gel extraction kit (Magen, China). Plasmids were also extracted and purified using kits from the same company. Purified PCR products and plasmids were sequenced (BGI, Shenzhen, China). The *aacC1* gene (0.543 kb) was amplified from plasmid pPR27 (**Table 1**) using primers Gm-f and Gm-r (**Table 2**) while the 0.8 kb *tsr* gene was amplified from plasmid pIJ6902 (**Table 1**) using primers Tsr-f and Tsr-r (**Table 2**).

**Table 2:** List of DNA primers used in the study.

Primers	Nucleotide sequence (5′-3′) with enzyme sites underlined	Restriction enzyme
Gm-f	GGGAATTCAAGCTTCAT*ATG*CCGAGAGCTTGGCACC	*Nde*I
Gm-r	CCCAAGCTTCTGCAG*TTA*GGTGGCGGTACTTGG	*Pst*I
Tsr-f	CGGCTGCAG*ATG*ACTGAGTTGGACAC	*Pst*I
Tsr-r	CCCAAGCTTTCTAGA*TTA*TCGGTTGGCCGCG	*Xba*I
Tsr-f1	GAGTAAGCCGATAAGCGACA	
Tsr-r1	TCGAGACTTGACATAATGTC	

*Start and stop codon italicized, enzyme site underlined.*

### Construction of Shuttle Vector Containing *tsr* + *aacC1* Resistance Genes

**FIGURE 1:**
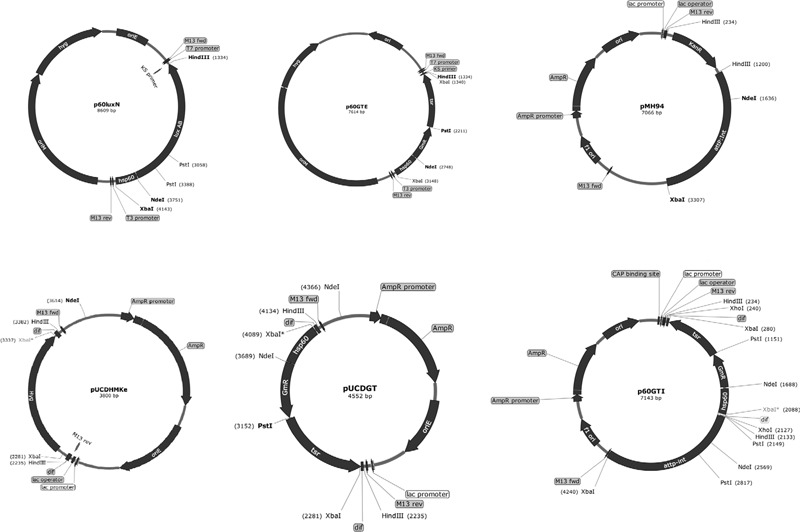
**Scheme of vector construction.** The *Escherichia coli*-mycobacterial plasmid p60GTE was derived by inserting *aacC1 and tsr* fragments next to the mycobacterial *hsp60* promoter. The *hsp60*-*aacC1-tsr* cassette was excised and inserted on to the *Xba*I sites of pUCDHmke bearing the *dif* sequences. The *dif*-*hsp60*-*aacC1-tsr-dif* cassette was excised and inserted on the *Hin*d III sites of plasmid pMH94. *attP*, mycobacteriophage L5 attachment site; *int*, integrase gene; oriE, origin region of *E. coli*; oriM, thermosensitive origin region of mycobacteria; KanR, KAN resistance gene, *dif*: the putative MTB *dif* sequence. Useful enzyme sites: *Nde*I; *Hin*dIII; *Pst*I and *Xba*I.

To construct a vector bearing *tsr*+*aacC1*, we arranged the genes into a cassette under the control of the *M. tuberculosis hsp60* promoter (**Figure 1**) in plasmid p60LuxN (Liu et al., 2015) intending the *aacC1* gene to be used for selection in *E. coli* and *M. smegmatis* mc^2^155 and the *tsr* gene to be used in *M. tuberculosis* and *M. bovis* BCG. The *aacC1* was cloned adjacent to the *hsp60* promoter into the *Nde*I-*Pst*I sites of p60LuxN resulting in plasmid p60Gm. The *tsr* gene was cloned into the *Pst*I-*Hin*dIII sites of plasmid p60Gm to get *E. coli*-mycobacteria shuttle plasmid p60GTE bearing *hsp60*-*aacC1-tsr* cassette.

### Construction of *dif*-*hsp60*-*aacC1*-*tsr*-*dif* Cassette

The *hsp60*-*aacC1-tsr* cassette was excised with *Xba*I from plasmid p60GTE and cloned into the *Xba*I sites of *E. coli* pUCDHmke derived from pTYdHm (Yang et al., 2014) plasmid (**Table 1**) bearing a *dif*-ΩHYG-*dif* cassette replacing *hyg* gene and creating plasmid pUCDGT. The *dif-hsp60*-*aacC1-tsr-dif* cassette (**Figure 1**) was excised by *Hin*dIII from pUCDGT and cloned into the integrative plasmid pMH94 (**Table 1**) replacing the KAN resistance gene and creating plasmid p60GTI.

### Transformation

Plasmids p60GTE and p60GTI were used. *M. smegmatis* was transformed as previously described (Snapper et al., 1990), while *M. tuberculosis* and *M. bovis* BCG were transformed as previously described (Wards and Collins, 1996; Yang et al., 2015) with some modifications. The competent *M. tuberculosis* and *M. bovis* BCG cells were first incubated at 37°C for 10 min before electroporation and transformation was performed at room temperature. Transformants were selected on plates containing TSR (5 and 10 μg/mL) for both *M. bovis* BCG and *M. tuberculosis* while containing GEN (5 μg/mL) for *M. smegmatis*. Individual transformant colonies of three independent transformations were counted to determine the transformation frequencies per microgram of DNA and tested by PCR with primers Tsr-f and Tsr-r.

### Analysis of Unmarked Recombinant *M. tuberculosis* and *M. bovis* BCG Transformants

Unmarked recombinant transformants were analyzed according to Yang et al. (2014). Briefly, PCR verified TSR-resistant single p60GTI colonies were individually cultured in 7H9 media to late log phase (OD_600_ = 0.8–1.0) without selection to allow excision of the *dif-hsp60*-*aacC1-tsr-dif* cassette by the endogenous mycobacteria XerC and XerD. Ten-fold serial dilutions of bacterial culture were spread on plain agar plates. The colonies were picked and replica streaked on both plain and 10 μg/mL TSR-containing 7H11 plates. The TSR-sensitive colonies were verified further by PCR amplification of the 1.9 kb cassette using primers Tsr-f1 and Tsr-r1 (**Table 2**) and the shorter PCR products (∼0.5 kb) bearing one single *dif* sequence were confirmed by sequencing.

## Results and Discussion

### TSR as a Potential Selection Antibiotic against Mycobacteria

We first tested the potential use of TSR as a selective antibiotic against mycobacteria. Using liquid culture autoluminescent *M. tuberculosis* H37Ra, we tested different TSR concentrations up to 10 μg/mL and the relative light units (RLUs) declined sharply within 2 days and continuously till the end of the assay, while those of blank control rose steadily (MIC_lux_ = 0.05 μM, ∼ = 0.08 μg/mL). Additional susceptibility testing on 2–50 μg/mL 7H11 TSR plates of *M. tuberculosis* and *M. bovis* BCG Tice yielded complete growth inhibition while we observed complete insensitivity even on 7H11 plates containing 100 μg/mL TSR for *M. avium, M. abscessus* GZ002 and *M. smegmatis* mc^2^155 illustrating the unsuitability of TSR as their selection antibiotic. We detected the TSR MICs of *M. tuberculosis* H37Rv strain and *M. bovis* BCG Tice as 0.125 and 0.25 μg/mL (**Table 3**) similar to the 0.08 μM (∼0.133 μg/mL to *M. tuberculosis* H37Rv) reported by Lougheed et al. (2009) and no mutant resistant colonies were observed.

**Table 3:** Minimum inhibition concentrations (MICs) of TSR for wild-type and recombinant mycobacteria.

*M. tuberculosis* and *M. bovis* BCG Tice strains	MIC (μg/mL)
*M. tuberculosis* H37Rv	0.125
*M. tuberculosis* H37Rv::p60GTE	>800
*M. tuberculosis* H37Rv::p60GTI	>800
*M. bovis* BCG Tice	0.25
*M. bovis* BCG Tice::p60GTE	160
*M. bovis* BCG Tice::p60GTI	160

### Construction of Plasmids p60GTE and p60GTI, Their Transformation Frequencies and MICs in Respective Recombinant Strains

We set out to construct two plasmids expressing *tsr* and *aacC1* genes in both *E. coli* and mycobacteria. We constructed episomal and integrative *E. coli*-mycobacterial shuttle plasmids bearing the mycobacterial *hsp60* promoter, *aacC1* and the *tsr* gene flanked by *dif* sequences (**Figure 1**). Both antibiotic resistance markers, the streptomyces TSR resistance gene, *tsr*, and the *Pseudomonas aeruginosa* GEN resistance gene, *aacC1*, worked in mycobacterial transformants. TSR resistance is not a selectable marker in *E. coli* due to outer membrane exclusion of TSR by gram-negative bacteria (Gale et al., 1981). To circumvent this, we used GEN for selection in *E. coli* and supplemented the media with chloramphenicol 170 μg/mL to increase the plasmid copy number. The transformation frequency for H37Rv and *M. bovis* BCG overexpressed with the episomal plasmid p60GTE were 1.26 × 10^4^ and 4.3 × 10^3^ CFUs and 3.5 × 10^3^ and 2 × 10^2^ CFUs, respectively, with the integrative plasmid p60GTI on TSR 5 μg/mL (**Table 4**). Both H37Rv and *M. bovis* BCG recombinant strains increased the MICs by >300-fold (**Table 3**) while *M. smegmatis* mc^2^155 strains increased the MICs by 40-fold (**Table 5**).

**Table 4:** Transformation frequency for *M. bovis* BCG Tice and *M. tuberculosis* H37Rv using TSR and *M. smegmatis* mc^2^155 using GEN as a selection marker.

	Transformation frequency for:		
Plasmids	*M. smegmatis*	*M. bovis*	*M. tuberculosis*
	mc^2^155	BCG-Tice	H37Rv
p60GTE	2.8 × 10^3^	4.3 × 10^3^	1.26 × 10^4^
p60GTI	1.5 × 10^3^	2 × 10^2^	3.5 × 10^3^

*Frequency represent the number of transformants per microgram of DNA.*

*Results are expressed as the mean number of colonies in triplicate experiments.*

**Table 5:** Minimum inhibition concentrations of GEN for wild-type and recombinant *M. smegmatis* mc^2^155.

*M. smegmatis* mc^2^155	MIC (μg/mL)
*M. smegmatis* mc^2^155	2.5
*M. smegmatis* mc^2^155::p60GTE	100
*M. smegmatis* mc^2^155::p60GTI	100

The loss of the *tsr* marker gene verified by PCR, yielded ∼0.5 kb products confirmed by sequencing to bear one *dif* sequence as expected, from 12 and 20 randomly selected recombinant p60GTI containing *M. tuberculosis* H37Rv and *M. bovis* BCG colonies. We found that five of each recombinant strain had lost the *tsr* gene which should be excised by the endogenous mycobacterial recombinase XerCD system expressed by XerC and XerD genes recognizing the Ω*dif* cassette (Cascioferro et al., 2010; Yang et al., 2014), resulting in selectable marker-free colonies.

Our TSR MICs results concurs with the antimicrobial bactericidal activity reported by others (Vermeulen and Wu, 2004; Lougheed et al., 2009), and to the best of our knowledge this is the first report showing the use of TSR resistance as putative selective marker for gene transfer in mycobacteria.

## Conclusion

We have successfully constructed a cassette containing *tsr* and *aacC1* genes flanked by *dif* sequences for selection in mycobacteria and demonstrated the potential of this cassette for use as a mycobacteria selection marker in *M. tuberculosis* and *M. bovis* BCG. The novelty of this work is the introduction and expression of genes in a new cassette and verified by raising of resistance in the corresponding host cells. The new reliable selection marker comes in handy for *M. tuberculosis* genetic manipulation studies and is a new tool for efficient construction of selection-marker free recombinant strains.

## Author Contributions

Conceived and designed research: JM, BW, YC, YZ, and TZ. Performed research: JM, GM, YC, SH, and CC. Co-wrote the manuscript: JM, GM, MN, TZ, YZ, and ST. Contributed reagents/materials and laboratory space for conducting mycobacterial experiments: TZ, YT, XL, JL, YZ, JD, and ST.

## Conflict of Interest Statement

The *dif-hsp60-aacC1-tsr-dif* cassette was filed as a patent for TZ, JM, BW, YC, SH, GM, YZ, and CC. The other authors declare that the research was conducted in the absence of any commercial or financial relationships that could be construed as a potential conflict of interest.
